# Impact of fertility treatments on perinatal outcomes in pregnancies complicated by GDMA1: a comparative cohort study

**DOI:** 10.1007/s00404-025-08183-4

**Published:** 2025-09-08

**Authors:** Tamar Eshkoli, Tal Atzmon, Reut Rotem, Adi Y. Weintraub, Yael Baumfeld, Naama Steiner

**Affiliations:** 1 Faculty of Health Sciences, Ben-Gurion University of the Negev, Beer‐Sheva, Israel; 2 Department of Obstetrics and Gynecology, Soroka University Medical Center, Beer‐Sheva, Israel; 3 Department of Obstetrics and Gynecology, Shaare Zedek Medical Center, Faculty of Medicine, Hebrew University of Jerusalem, Jerusalem, Israel

**Keywords:** Gestational diabetes mellitus (GDM), gdma1, Fertility treatment, Ovulation induction (OI), Assisted reproductive technology (ART), In vitro fertilization (IVF), Pregnancy complications

## Abstract

**Objective:**

To investigate adverse pregnancy and delivery outcomes in women with GDMA1 during pregnancies conceived through fertility treatments.

**Methods:**

This population-based retrospective cohort study examined adverse pregnancy and delivery outcomes in pregnancies affected by GDMA1 following fertility treatments compared to those conceived naturally. Women with GDMA1 who conceived via fertility treatments were classified as cases, while those who conceived naturally were designated as controls. Multivariable logistic regression models were employed to evaluate the association between fertility treatments and perinatal outcomes in pregnancies complicated by GDMA1.

**Results:**

The study cohort consisted of 12,841 pregnant women diagnosed with GDMA1, of whom 640 (4.98%) conceived through fertility treatments, while the remaining 12,201 conceived naturally. Women with GDMA1 who conceived through fertility treatments had significantly higher rates of preeclampsia, hypertension, preterm birth, and cesarean delivery, while they experienced lower rates of macrosomia and polyhydramnios compared with women who conceived spontaneously. Multivariate analysis identified fertility treatments as an independent predictor of cesarean delivery, while conversely serving as a protective factor against macrosomia after adjusting for maternal age, parity, hypertensive disorders of pregnancy, and labor induction.

**Conclusion:**

Women with GDMA1 who conceived through fertility treatments experienced a reduced incidence of complications associated with GDM, such as macrosomia and polyhydramnios. This could be explained by better GDM control and adherence to a more rigorous prenatal care approach. However, they faced an increased risk of other obstetric complications, including pre-eclampsia, preterm delivery, and cesarean delivery.

## Introduction

Gestational diabetes mellitus (GDM) is the most prevalent metabolic disorder during pregnancy, defined as glucose intolerance first detected in the second or third trimester [1]. GDM is classified into GDMA1, controlled with diet and exercise, and GDMA2, requiring insulin or oral hypoglycemics [2]. Its prevalence is rising worldwide, now affecting − 6% of pregnancies [3]. Women with GDM are at increased risk of hypertensive disorders, cesarean delivery, and neonatal complications including macrosomia, hypoglycemia, and hyperbilirubinemia [4, 5]. Lifestyle interventions have been shown to reduce adverse outcomes, particularly large-for-gestational-age (LGA) infants and macrosomia [5].

Assisted reproductive technologies (ART) include ovulation induction (OI) and in vitro fertilization (IVF) [6, 7].

ART pregnancies are consistently linked with higher rates of obstetric complications compared with natural conception [8]. Prior studies have reported increased risks of preterm birth, hypertensive disorders, cesarean delivery, and neonatal morbidity among ART pregnancies, including those complicated by GDM [9–13].

However, limited data exist specifically on women with GDMA1. On one hand, ART pregnancies are associated with increased obstetric and perinatal risks. On the other hand, women with GDMA1 undergoing fertility treatments may achieve better glycemic control due to closer medical surveillance. We focused on GDMA1 to maintain a homogenous study population, avoiding heterogeneity introduced by insulin-treated cases (GDMA2), which represent a different severity and management pattern of GDM. Our study aimed to evaluate whether fertility treatments influence pregnancy and delivery outcomes in this distinct population.

## Materials and methods

### Study design

This research was conducted as a retrospective cohort study using a population-based approach, that compares women with GDMA1 who conceived through fertility treatments (study group) with those who conceived naturally (control group).

Data was identified through the Soroka University Medical Center )SUMC( perinatal database. The patient demographic**,** clinical, and obstetrical data, including delivery and perinatal outcomes, are documented immediately after delivery by the attending obstetricians and subsequently reviewed by experienced medical secretaries before being input into the database to ensure optimal accuracy and comprehensiveness.

The medical records contain information about clinical and demographical features (maternal age, ethnicity, gravidity, and parity), current pregnancy and delivery (GDMA1 or non-diabetic, fertility treatment subtype, delivery gestational age, and delivery method), and adverse perinatal outcomes. The primary outcomes examined were pregnancy complications and adverse perinatal outcomes.

### Settings

The study was carried out at the Soroka University Medical Center (SUMC), a major teaching hospital and the sole tertiary medical facility in the region. Serving a population of over one million people and recording more than 15,000 births annually, SUMC provides non-selective, population-based data. All deliveries are managed under the same standard of care, ensuring consistent management for both groups at delivery.

The study was approved by the institutional ethics committee (SUMC IRB Committee #0458-20-SOR, November 9, 2020).

### Study population

All singleton pregnancies of women with GDMA1 who delivered at the SUMC between the years 1996 and 2016 were included. The study included GDMA-1 pregnancies with singleton deliveries in women aged 18 and older. Exclusion criteria encompassed pregnancies in women with pre-gestational diabetes mellitus, those complicated by GDMA-2, pregnancies with known chromosomal abnormalities or major malformations, and cases lacking prenatal care.

Definitions of study outcomes were as follows: macrosomia was defined as a suspected fetal weight > 4000 g; small for gestational age (SGA) was defined as birth weight below the 10th percentile for gestational age; large for gestational age (LGA) was defined as birth weight above the 90th percentile for gestational age; and postpartum hemorrhage (PPH) was defined as an estimated maternal blood loss ≥ 1000 mL within 24 h after delivery.

### Statistical analysis

The data collected were coded and stored using Microsoft Excel and analyzed with SPSS version 23.0 (SPSS, Chicago, IL). Descriptive statistics, including mean, standard deviation (SD), and graphs, were initially used, followed by advanced statistical analyses with various parametric tests. Continuous variables with a normal distribution (e.g., maternal age) were expressed as mean ± SD and compared between the groups using the t-test. Non-normally distributed continuous variables (e.g., parity, gravidity) were presented as median with interquartile range and analyzed using the Mann–Whitney test. Categorical variables were described as counts and percentages, with statistical analysis conducted using the Chi-Square test or Fisher's Exact test, as appropriate (e.g., ART). A two-sided p-value of < 0.05 was considered statistically significant.

To evaluate whether pregnancies conceived through fertility treatments in women with GDMA1 are independently associated with adverse delivery outcomes, a multivariate regression model was developed. Dependent variables were included in the model if their prevalence differed significantly between the study groups.

## Results

Among the 12,841 women diagnosed with GDMA1 included in the final analysis, 640 pregnancies (4.98%) resulted from fertility treatments, while the remaining 12,201 were conceived naturally.

Patient demographics, baseline characteristics, and pregnancy and delivery outcomes are presented in Tables 1, 2 and 3. Patients with GDMA1 who underwent fertility treatments were significantly older (33.34 ± 5.70 vs 31.93 ± 5.80, P < 0.001) and the percentage of Bedouin ethnicity patients among this group was significantly lower (15.6% vs 36.7%, P < 0.001). A higher prevalence of nulliparity was observed in the study group (57.7% vs 19.7%, P < 0.001). Among the control group, there was a notably higher rate of patients with a previous cesarean Sect. (14.1% vs 17.8%, P = 0.02).

**Table 1:** Maternal demographic and baseline characteristics of women with GDMA1 by mode of conception

Variables	Fertility tx^1^ N = 640	No fertility tx n = 12201	P value
Age (years)	33.34 ± 5.70	31.93 ± 5.80	< 0.001
Ethnicity			
Jew	540 (84.4)	7720 (63.3)	
Bedouin	100 (15.6)	4481 (36.7)	< 0.001
Nulliparity	369 (57.7)	2405 (19.7)	< 0.001
Gravidity	2.33 ± 1.69	4.85 ± 3.45	< 0.001
Parity	1.63 ± 0.90	4.11 ± 3.00	< 0.001
S/P^2^ CS^3^	90 (14.1)	2166 (17.8)	0.02

^1^tx: treatments

^2^S/P: status post

^3^CS: caesarean section

**Table 2:** Pregnancy complications among women with GDMA1 by mode of conception

Variables	Fertility tx^1^ N = 640	No fertility tx n = 12201	P value
HTN^4^			
All	100 (15.6)	1280 (10.5)	< 0.001
PET^5^	69 (10.8)	827 (6.8)	< 0.001
Eclampsia	0 (0.0)	3 (0.0)	1.00
Mild	52 (8.1)	630 (5.2)	0.001
Severe	17 (2.7)	196 (1.6)	0.04
Polyhydramnios	38 (5.9)	1178 (9.7)	0.01
Macrosomia	20 (3.1)	1201 (9.8)	< 0.001
Previa			
Placenta	13 (2.0)	64 (0.5)	< 0.001
Vasa	1 (0.2)	9 (0.1)	0.40
Induction of labor	275 (43.0)	4742 (38.9)	0.04
Abruption	6 (0.9)	82 (0.7)	0.45
Preterm Delivery	76 (11.9)	923 (7.6)	< 0.001

^1^tx: treatments

^2^S/P: status post

^3^CS: caesarean section

^4^HTN: hypertension

^5^PET: pre-eclampsia toxemia

**Table 3:** Delivery and neonatal outcomes among women with GDMA1 by mode of conception

Variables	Fertility tx^1^ N = 640	No fertility tx n = 12201	P value
Gestational age (weeks)	38.19 ± 1.75	38.82 ± 1.71	< 0.001
Mode of delivery			
PS^6^	359 (56.1)	8829 (73.2)	< 0.001
Vacuum	20 (3.1)	369 (3.0)	
CS	261 (40.8)	2903 (23.8)	
PPH^7^	9 (1.4)	77 (0.6)	0.04
AGA^8^	581 (90.8)	10,581 (86.7)	
LGA^9^	27 (4.2)	1324 (10.9)	< 0.001
SGA^10^	32 (5.0)	296 (2.4)	< 0.001
Shoulder dystocia	0 (0.0	46 (0.4)	0.17
Apgar			
1 min	8.57 ± 1.25	8.69 ± 1.07	0.02
5 min	9.85 ± 0.67	9.90 ± 0.53	0.07
< 7 score	6 (0.9)	58 (0.5)	0.14
Perinatal mortality			
All	2 (0.3)	57 (0.5)	1.00
Neonatal gender			
Male	325 (50.8)	6424 (52.7)	0.36
Female	315 (49.2)	5777 (47.3)	

^1^tx: treatments

^2^S/P: status post

^3^CS: caesarean section

^4^HTN: hypertension

^5^PET: pre-eclampsia toxemia

^6^PS: partus spontaneous

^7^PPH: postpartum hemorrhage

^8^AGA: appropriate for gestational age

^9^LGA: large for gestational age

^10^SGA: small for gestational age

Patients with GDMA1 who underwent fertility treatment had significantly higher rates of various pregnancy complications such as hypertension (15.6% vs 10.5%, P < 0.001), preeclampsia (10.8% vs 6.8%, P < 0.001), placenta previa (2.0% vs 0.5%, P < 0.001), induction of labor (43.0% vs 38.9%, P = 0.04) and preterm birth (11.9% vs 7.6%, P < 0.001). On the other hand, patients who conceived naturally had higher rates of GDM-related complications such as polyhydramnios (9.7% vs 5.9%, P = 0.01) and macrosomia (10.8% vs 5.2%, P < 0.001). No significant differences were found between the two groups regarding placental abruption.

Regarding delivery outcomes, patients in the study group had significantly higher rates of cesarean deliveries (40.8% vs 23.8%, P < 0.001), vacuum-assisted deliveries (3.1% vs 3.0%, P < 0.001), and post-partum hemorrhage (1.4% vs 0.6%, P = 0.04).

A higher percentage of neonates in the study group were smaller for gestational age (5.0% vs 2.4%, P < 0.01). Their Apgar score was significantly lower in the 1st minute (8.57 ± 1.25 vs 8.69 ± 1.07, P = 0.02) but not in the 5th minute (9.85 ± 0.67 vs 9.90 ± 0.53, P = 0.07). No significant differences were found between the groups regarding shoulder dystocia and perinatal mortality.

In the multivariate analysis assessing the likelihood of cesarean section (Table 4), women with GDMA1 who conceived via fertility treatments showed an elevated risk for cesarean delivery after adjusting for maternal age, parity, hypertensive disorders of pregnancy, and labor induction (40.8% vs. 23.8%; OR = 2.22, 95% CI = 2.09–2.35, P < 0.001). On the other hand, a separate multivariate analysis for macrosomia prediction (Table 5) revealed that fertility treatments served as a protective factor for macrosomia after accounting for maternal age, parity, hypertensive disorders of pregnancy, and labor induction (3.1% vs. 9.8%; OR = 0.83, 95% CI = 0.73–0.94, P < 0.001).

**Table 4:** Multivariate analysis for the prediction of CS in patients with GDMA1 who conceived after fertility treatments

Variables	OR	95% CI	P value
Age	1.09	1.08–1.09	< 0.001
Hypertension	2.54	2.44–2.64	< 0.001
Induction	0.58	0.57–0.60	< 0.001
Nulliparity	1.77	1.72–1.82	< 0.001
Macrosomia	1.76	1.69–1.84	< 0.001
Fertility	2.22	2.09–2.35	< 0.001

**Table 5:** Multivariate analysis for the prediction of macrosomia in patients with GDMA1 who conceived after fertility treatments

Variables	OR	95% CI	P value
Age	1.06	1.06–1.06	< 0.001
Hypertension	1.12	1.04–1.21	0.01
Induction	1.10	1.05–1.15	< 0.001
Grandmultiparity	1.46	1.39–1.52	< 0.001
Fertility	0.83	0.73–0.94	0.01

## Discussion

Our population-based study showed that among women with GDMA1, those who conceived after fertility treatments had higher risks of obstetrics complications, including hypertension, pre-eclampsia, placenta previa, cesarean section, induction of labor, post-partum hemorrhage, preterm delivery, and small for gestational age infants. Conversely, they had lower risk of diabetes-related complications such as polyhydramnios and macrosomia. In multivariate analysis, fertility treatments remained an independent predictor of cesarean section, while simultaneously acting as a protective factor against macrosomia, after adjusting for maternal age, parity, hypertensive disorders of pregnancy, and labor induction.

Our results align with previous research indicating an elevated risk of pregnancy complications in the fertility treatment group. Pandey et al. [8] demonstrated that singleton pregnancies conceived through ART are linked to a higher likelihood of obstetric and perinatal complications compared to naturally conceived pregnancies [8]. Comparable results were found in studies by Kouhkan et al. [9] and Qin et al. [10]. Furthermore, higher cesarean rates were reported following ART [9, 10]. In our study, the cesarean rate in the ART group was approximately 40%, which is consistent with previous literature. This elevated rate likely reflects higher maternal age, previous uterine surgery, and obstetric complications, rather than differences in prenatal care or fetal size [14]. Herman et al. [13] and Harlev et al. [11] also reported increased rates of pre-eclampsia and cesarean delivery in ART pregnancies.

Not all of our findings matched prior studies. Pandey et al. [8] and Qin et al. [10] showed a higher risk for perinatal mortality in the fertility treatments group, while in our study the difference was not significant, possibly due to our exclusion of pregnancies with known chromosomal abnormalities and major malformations. Moreover, our study included only women with GDMA1, whereas prior studies included a broader population [8, 10]. Qin et al. [10] reported higher rates of polyhydramnios, whereas in our study the risk was lower, and placental abruption did not differ significantly.

The lower rate of macrosomia observed in our study (approximately three times lower in the study group) may be explained by more frequent prenatal follow-ups and better glucose control among women with GDMA1 undergoing fertility treatments. Multivariate analysis confirmed that fertility treatments significantly reduced macrosomia risk after adjusting for confounders. Overall, the combination of fertility treatments and GDMA1 likely contributed to earlier diagnosis and management of pregnancy complications, and explains the elevated cesarean rate in ART pregnancies.

The strengths of this study include the use of the SUMC database, the only tertiary hospital in southern Israel, which serves the entire local population. In Israel, all residents are covered for health expenses, including pregnancy care and delivery, and most are likely to give birth at this hospital, ensuring a non-selective study population. Additionally, the data was obtained from computerized records, which were entered by obstetricians immediately after delivery, minimizing recall bias. The inclusion of IVF patients with GDM, a clinically significant group expected to grow in the coming years, is another notable strength of this study.

Our study has also some limitations. First is its retrospective design, which may demonstrate an association, but not causation. On the other hand, the power of our findings is robust due to the large sample size. In addition, a retrospective database holds the possibility of missing data. However, the immediate documentation by an obstetrician after delivery, coupled with the review of the data by experienced medical secretaries before it is entered into the database, ensures high accuracy and thoroughness while minimizing the risk of errors.

In conclusion, pregnant women with GDMA1 who conceived through fertility treatments are at an increased risk for obstetric complications such as pre-eclampsia, preterm birth, and cesarean delivery. However, they experience lower rates of diabetes-related complications like macrosomia and polyhydramnios, likely due to better GDM control and a more rigorous prenatal care, as well as early diagnosis and management of pregnancy complications.

## Data Availability

No datasets were generated or analysed during the current study.
